# Relationship Between Occupational Identity and Work Engagement Among Professional Caregivers: The Mediating Role of Psychological Capital

**DOI:** 10.1155/jonm/9660875

**Published:** 2026-10-18

**Authors:** Linqi Mo, Yueyue He, Rui Wang, Weixiu Ouyang, Ling Feng

**Affiliations:** ^1^ Department of Neurology West China Hospital Sichuan University/West China School of Nursing Sichuan University Chengdu China scu.edu.cn

**Keywords:** occupational identity, professional caregiver, psychological capital, work engagement

## Abstract

**Objective:**

The theoretical basis of this research is the Job Demands–Resources (JD‐R) model and the Conservation of Resources (COR) theory. This research aims to explore the mediating role of psychological capital in the relationship between occupational identity and work engagement among professional caregivers.

**Backgrounds:**

Amidst China’s evolving social landscape, driven by a rapidly aging population and the implementation of ‘unaccompanied ward’ policies, hospitals are progressively professionalizing and standardizing nursing tasks previously performed by family members. This shift has triggered a surge in demand for professional caregivers. However, the stability of this workforce is severely undermined by a confluence of systemic and occupational challenges, which collectively hinder improvements in service quality.

**Methods:**

This cross‐sectional survey was conducted in Sichuan Province, China, from January to February 2024. Data were collected via the Occupational Identity Scale (OIS), Utrecht Work Engagement Scale (UWES), and Psychological Capital Questionnaire (PCQ‐24). The study was conducted among professional caregivers, and 296 valid questionnaires were included in the analysis. Mediation analysis was performed via structural equation modeling. Indirect effects were evaluated through bootstrapping. Data analysis was performed using the statistical programs SPSS 26.0 and AMOS 24.0.

**Results:**

Professional caregivers’ occupational identity was positively related to work engagement (*r* = 0.667, *p* < 0.001) and psychological capital (*r* = 0.574, *p* < 0.001). Professional caregivers’ work engagement was also positively related to their level of psychological capital (*r* = 0.613, *p* < 0.001). Psychological capital played a partial mediating role in the association between occupational identity and work engagement, and the mediating effect was 1.147 (95% CI [0.758, 1.682]), accounting for 36.42% of the total effect.

**Conclusion:**

Our findings reveal that occupational identity is positively associated with work engagement among professional caregivers, both directly and indirectly through its link with psychological capital. This suggests that psychological capital may play a meaningful role in the connection between occupational identity and work engagement. Therefore, efforts to strengthen both occupational identity and psychological resources may be relevant for supporting workforce engagement. To promote higher work engagement among professional caregivers, managers may consider developing psychological capital alongside ensuring adequate professional resources and security.

**Implication for Nursing Management:**

Given that professional caregivers’ work commitment is intrinsically linked to the quality of patient care, nursing managers are encouraged to recognize professional caregivers as pivotal stakeholders in the delivery of safe care. By integrating existing strategies that ensure adequate professional resources and job security, managers may bolster caregivers’ occupational identity and psychological capital. Such an approach holds the potential not only to fortify workforce stability but also to empower employees to engage proactively and autonomously in patient care, ultimately safeguarding patient safety. These findings offer specific suggestions for nursing management practices.

## 1. Introduction

According to the 2020 China Population Census of China [1], the nation’s population growth rate has declined steadily since 1982, accompanied by a rapid increase in the population aged 60 and above, marking China’s transition into an aging society. Concurrently, shrinking family sizes, the waning of multigenerational coresidence, and the dispersion of family members due to rapid urbanization and rural‐to‐urban migration have severely strained traditional family caregiving capacities [2]. Consequently, an increasing number of hospitalized patients are facing the situation of being left unattended. Furthermore, amidst China’s ongoing medical reforms—specifically the promotion of ‘unaccompanied’ ward policies—hospitals are progressively transforming nursing tasks previously performed by families into professionalized and standardized services. In this evolving landscape, professional caregivers have emerged as a critical component of the healthcare system, driving the rapid expansion of specialized caregiver agencies.

These agencies, authorized by hospitals, provide unified management for caregivers who are temporarily hired by patients or their families. Their duties extend beyond basic activities of daily living (ADLs)—such as bed‐making, feeding, bathing, hygiene care, and suctioning—to include medication administration, health information dissemination, communication facilitation, and emotional support during hospitalization [3].

However, a significant supply‐demand gap persists. Surveys indicate that while China requires over 10 million professional caregivers, the current workforce numbers fewer than 1 million [4]. Despite their indispensability, professional caregivers face a confluence of systemic and occupational challenges: limited social support, societal stigma, low wages, inadequate benefits, high work intensity (e.g., long hours, heavy physical loads, and exposure to infectious diseases), and significant emotional labor associated with caring for the terminally ill. These factors collectively contribute to high psychological stress, low job satisfaction, and excessive turnover [5, 6].

This workforce instability severely hinders the improvement of service quality. Conversely, a stable and highly engaged caregiver team can reduce nursing errors, enhance patient satisfaction, mitigate doctor‐patient conflicts, and promote the sustainable operation of the medical system. Yet, most existing management strategies remain overly focused on material incentives, often overlooking the pivotal role of individual psychological resources in fostering career persistence.

Psychological capital refers to a positive psychological state that an individual exhibits during their growth and development, which is specifically manifested as follows: (1) self‐efficacy: having confidence and being able to make necessary efforts to achieve success when facing challenging work; (2) optimism: having a positive attitude toward current and future success; (3) hope: being able to achieve the goal and be able to adjust the approach to reach it when necessary for success; and (4) resilience: sustaining effort and bouncing back quickly from adversity or problems to attain success. Work engagement is defined as “a positive, fulfilling, work‐related state of mind characterized by vigor, dedication, and absorption” [7]. Occupational identity refers to the recognition and acceptance of professional roles given by society. It also refers to a positive response to the aim of one’s career, social value, and other factors [8].

Grounded in the broader positive psychology movement and specifically within positive organizational behavior, we posit that cultivating positive psychological capabilities—such as optimism, hope, and self‐efficacy—fosters desirable employee outcomes, including strengthened professional identity and enhanced work engagement [9]. The job demands–resources (JD‐R) model proposes that working conditions can be categorized into 2 broad categories: job demands and job resources [10, 11]. According to the JD‐R model, job demands in the working environment (such as emotional stress and physical exertion) consume individual resources and lead to burnout. Work resources (such as social support and autonomy) and individual resources (such as psychological capital and professional identity) can stimulate work engagement and enhance performance.

Complementing this, Conservation of Resources (COR) theory posits that individuals strive to acquire, protect, and accumulate resources. When facing resource loss threats, individuals mobilize existing resources to resist depletion. For professional caregivers, core resources include professional dignity, social recognition, technical competence, and psychological energy. Threats to these resources—such as diminished occupational identity or an imbalance where high job demands outweigh available resources—can precipitate burnout and reduced work engagement. Conversely, individuals with abundant psychological capital are better equipped to cope with challenges, safeguard existing resources, and generate new ones, thereby creating a spiral effect that further enhances work engagement [12, 13].

Although current studies have revealed relationships between professional identity and work engagement [14], psychological capital and work engagement [15], and occupational identity and psychological capital [7], these studies have predominantly focused on teachers, physicians, and registered nurses. Research targeting professional caregivers remains scarce. In China’s hierarchical healthcare system, tertiary hospitals represent the highest tier of medical care, shouldering the most critical and complex clinical cases, as well as teaching and research responsibilities. Professional caregivers in these settings are exposed to exceptionally high workloads, intense emotional labor, and complex interpersonal demands. Given the severe workforce instability among professional caregivers and the limitations of existing material‐incentive‐focused management strategies, investigating occupational identity and work engagement within this high‐pressure environment is of critical importance for management. Therefore, this study aims to explore whether the psychological capital of professional caregivers has a mediating effect on the relationship between occupational identity and work engagement. Our findings will provide a theoretical foundation and practical implications for enhancing caregivers’ career development, mental health, and work efficiency, ultimately contributing to improved service quality and greater social recognition for the profession.

## 2. Methods

### 2.1. Study Design

This was a cross‐sectional survey carried out in Sichuan Province, China, between January and February 2024. Data were collected using a structured questionnaire administered by the research team.

### 2.2. Ethical Considerations

This study was conducted in accordance with the ethical principles of the Declaration of Helsinki (1964), and it was approved by the Ethics Committee of West China Hospital, Sichuan University (Approval NO. 2022[1969]). Electronic informed consent was obtained from all participants prior to their participation.

### 2.3. Data Collection

The structured questionnaire was hosted on Wenjuanxing (a widely used online survey platform in China). The survey link was distributed to eligible professional caregivers via WeChat, a popular social media application. To ensure data quality, the platform settings required respondents to complete all mandatory items before submission. The first page of the online questionnaire displayed the study information and an electronic informed consent form; participants indicated their consent by proceeding to the survey items. To prevent duplicate responses, the system restricted submissions to one per account and IP address. Due to the anonymous nature of the online survey, the exact number of potential participants who viewed the invitation but declined was not recorded. However, we implemented strict data cleaning procedures to ensure quality.

### 2.4. Participants

From January to February 2024, convenience sampling was used to select third‐party contracted professional caregivers of tertiary hospitals in Sichuan Province, China.

The subjects were enrolled if they met the following criteria: (1) were ≥ 18 years old; (2) currently employed as a full‐time clinical caregiver on duty; (3) had ≥ 1 year of working experience; (4) voluntary participation with signed informed consent; and (5) could read and complete the questionnaire.

The exclusion criteria were as follows: (1) individuals on extended leave (e.g., sick leave) during the data collection period; (2) those suspended from duty or who declined to participate; (3) regular respondent (e.g., all the answers chose the same option); and (4) completion time < 600 s.

### 2.5. Sample Size

To determine the minimum sample size required to detect the mediation effect in the main study, an a priori power analysis was conducted using the Monte Carlo simulation method [16]. The analysis was based on the observed correlations and standard deviations from a pilot study and was performed using the web application developed by the authors. The specific parameters were set as follows: the correlation between Psychological Capital and Occupational Identity was *r* = 0.232, between Psychological Capital and Work Engagement was *r* = 0.602, and between Occupational Identity and Work Engagement was *r* = 0.484. The standard deviations were SD = 10.894 for Psychological Capital, SD = 5.448 for Occupational Identity, and SD = 8.459 for Work Engagement. For the simulation, the significance level (*α*) was set at 0.05, the desired statistical power (1−*β*) at 0.80, and the confidence interval (CI) at 95%, with 5000 Monte Carlo draws. The results indicated that a minimum sample size of 203 was required to detect the expected mediation effect. To account for potential invalid responses and sample attrition, we oversampled during the formal data collection phase to ensure adequate statistical power. A total of 314 questionnaires were initially collected. Following a rigorous data cleaning process conducted by two independent nurse researchers, 18 invalid responses were excluded, resulting in a final analytic sample of 296 valid questionnaires (response rate: 94.3%). This final sample size exceeds the minimum requirement suggested by the a priori power analysis (296 > 203), indicating that the study possesses sufficient statistical power to test the research hypotheses.

### 2.6. Measures

The questionnaire was written in Chinese and consisted of four parts, including the following four subsections: the Demographic questionnaire, the Occupational Identity Scale (OIS), the Psychological Capital Questionnaire (PCQ‐24) and the Utrecht Work Engagement Scale (UWES‐9).

### 2.7. Demographic Questionnaire

The demographic characteristics included sex, age, years of work experience, education level, marital status, monthly income, position, level of work unit, working ward, and whether professional training had been received.

### 2.8. OIS

The OIS designed by Tyler and McCallum [17] was used to measure the occupational identity level of professional caregivers. This study adopted the Chinese version revised by Cai Changjuan of Chaoyang University of Technology, Taiwan Province of China. The Chinese version of the OIS has been widely used in the Chinese population and has demonstrated satisfactory reliability [18, 19]. The scale consists of 10 items scored on a 5‐point Likert scale (ranging from 1 = completely inconsistent to 5 = completely consistent); the scale score ranges between 10 and 50 points, and the higher the score is, the higher the level of occupational identity. In the present survey, the Cronbach’s *α* for the total scale was 0.906, and the split‐half reliability was 0.882, indicating that the questionnaire had satisfactory reliability. The KOM value of this questionnaire is 0.912, and the *X* ^2^ of Bartlett’s sphere is 1626.195, which is significant, indicating that the questionnaire has satisfactory validity.

### 2.9. PCQ‐24

The PCQ‐24, designed by Luthans et al., is used to measure the psychological capital (PsyCap) of employees [20]. It consists of four dimensions: self‐efficacy, hope, resilience, and optimism. The total scale consists of 24 items, and each of the four dimensions is measured by six items. Each of the items is scored on a six‐point Likert scale ranging from 1 (strongly disagree) to 6 (strongly agree). All the questions asked the participants how they feel “right now.” A higher score indicated a higher level of PsyCap. The PCQ‐24 has demonstrated adequate reliability and construct validity across multiple samples [20]. The Chinese PCQ‐24 has been widely validated and applied among Chinese occupational groups [21–23]. In the present study, the Cronbach’s *α* for the total scale was 0.860, and the split‐half reliability was 0.810, indicating that the questionnaire had satisfactory reliability. The KOM value of this questionnaire is 0.902, and the *X* ^2^ of Bartlett’s sphere is 2944.285, which is significant, indicating that the questionnaire has satisfactory validity.

### 2.10. UWES‐9

Schaufeli and Bakker developed the UWES‐9 [24], which aims to measure the concept of work engagement. Work engagement is defined as a positive, fulfilling, work‐related state of mind [25]. The scale contains nine items in total (3 items per subscale), rated on a seven‐point Likert scale ranging from 0 (“never”) to 6 (“every day”). Vigor is characterized by high levels of energy and mental resilience while one works and by the willingness and ability to invest effort in one’s work. Dedication is characterized by a sense of significance, enthusiasm, inspiration, pride, and challenge. A final scale that is distinguished is absorption, which is characterized by being fully concentrated and happily engrossed in one’s work [26]. The Chinese version has been shown to be reliable [25]. In the present study, the Cronbach’s *α* for the total scale was 0.907, and the split‐half reliability was 0.881, indicating that the questionnaire had satisfactory reliability. The KOM value of this questionnaire is 0.921, and the *X* ^2^ of Bartlett’s sphere is 1413.680, which is significant, indicating that the questionnaire has satisfactory validity.

### 2.11. Statistical Analysis

Data analysis was conducted via the statistical programs SPSS 26.0 and AMOS 24.0. First, the participants’ general characteristics were analyzed via descriptive statistics. Second, Pearson’s correlation coefficient was used to analyze the correlations among psychological capital, occupational identity, and work engagement. Third, AMOS 24.0 was used to construct a structural equation model (SEM) of the mediating role of psychological capital between occupational identity and work engagement. Occupational identity and psychological capital are the independent variables, and work engagement is the dependent variable. In addition, subscale scores are utilized as indicators for the latent factor. Bootstrapping was used to test the significance of the mediation effects. We ran 5000 bootstrap resamples, adopting 95% CIs to test both direct and indirect effects. If the 95% CI of the indirect effect did not include zero, the mediation effect was considered statistically significant [27]. The fit between the hypothetical model and the data were examined by calculating the absolute and incremental fit indices. A good model fit was indicated by the following criteria: a chi‐square to degrees of freedom (CMIN/DF) < 3 ratio, a root mean square error of approximation (RMSEA) < 0.05 or at least 0.08, and normed fit index (NFI), Tucker‒Lewis index (TLI), and comparative fit index (CFI) > 0.90 [28]. A *p* value < 0.05 was considered to indicate statistical significance. Furthermore, given the reliance on self‐reported measures, the potential for common method bias (CMB) was addressed by employing Harman’s single‐factor test. Specifically, if the first unrotated factor accounts for less than 40% of the total variance, no severe CMB is indicated [8]. In this study, the first unrotated principal component accounted for 34.40% of the total variance, which was below the 40% threshold, suggesting that CMB was not a serious concern in the present study.

## 3. Results

### 3.1. Participant Characteristics

A total of 58 (19.6%) male and 238 (80.4%) female professional caregivers participated in the study, with average ages of 51.45 ± 6.19 years for women and 52.67 ± 6.19 years for men. The general characteristics of the patients are depicted in Table 1.

**TABLE 1:** General characteristics (*N* = 296).

Variable	Category	*N* (%)
Gender	Male	58 (19.6)
	Female	238 (80.4)

Education	Primary school or lower	59 (19.9)
	Junior high school	177 (59.8)
	Senior high school/secondary specialized school	48 (16.2)
	Junior college	11 (3.7)
	Bachelor and above	1 (0.3)

Marital status	Married	260 (87.8)
	Unmarried	6 (2)
	Divorced	20 (6.8)
	Widowed	10 (3.4)

Having professional training in professional care	Yes	293 (99)
	No	3 (1)

Monthly income, CNY	≤ 3000	27 (9.1)
	3001–5000	188 (63.5)
	5001–8000	77 (26)
	> 8000	4 (1.4)

Position	Professional caregiver	255 (86.1)
	Team Leader	39 (13.2)
	Supervisor	2 (0.7)

Time as a professional caregiver	≤ 3 years	100 (33.8)
	> 3 years and ≤ 5 years	58 (19.6)
	> 5 years and ≤ 10 years	84 (28.4)
	> 10 years	54 (18.2)

Ward in which they worked	Medical	85 (28.7)
	Surgical	102 (34.5)
	Obstetrics & Gynecology	3 (1)
	Emergency	6 (2)
	Oncology	14 (4.7)
	Psychiatry	7 (2.4)
	Geriatrics	25 (8.4)
	Pediatric	2 (0.7)
	Others	52 (17.6)

### 3.2. Correlation Analysis

Correlation analysis was used to explore the relationships among professional caregivers’ occupational identity, psychological capital, work engagement, and the dimensions of each scale. As shown in Table 2, there were bivariate correlations between the subfactors of the main variables. Specifically, occupational identity was positively correlated with both work engagement (*r* = 0.667, *p* < 0.001) and psychological capital (*r* = 0.574, *p* < 0.001). Similarly, work engagement showed a strong positive association with psychological capital (*r* = 0.613, *p* < 0.001). All subscale scores were also positively intercorrelated. In addition, most of the coefficients were less than 0.80 (i.e., the standard for multicollinearity), indicating that there was no apparent multicollinearity problem.

**TABLE 2:** Correlations between psychological capital, occupational identity, and work engagement (*N* = 296).

Variables	Mean (SD)	1	1‐1	1‐2	1‐3	1‐4	2	3	3‐1	3‐2	3‐3
1. Psychological capital	117.05 (12.14)	1									
1‐1 Self‐efficacy	29.84 (3.99)	0.836^∗∗^	1								
1‐2 Hope	30.44 (3.76)	0.878^∗∗^	0.697^∗∗^	1							
1‐3 Resilience	28.84 (3.63)	0.840^∗∗^	0.591^∗∗^	0.682^∗∗^	1						
1‐4 Optimism	27.93 (3.47)	0.708^∗∗^	0.402^∗∗^	0.476^∗∗^	0.476^∗∗^	1					
2. Occupational identity	41.68 (6.43)	0.574^∗∗^	0.469^∗∗^	0.612^∗∗^	0.506^∗∗^	0.275^∗∗^	1				
3. Work engagement	41.18 (9.63)	0.613^∗∗^	0.521^∗∗^	0.625^∗∗^	0.527^∗∗^	0.319^∗∗^	0.667^∗∗^	1			
3‐1 Vigor	13.75 (3.47)	0.630^∗∗^	0.536^∗∗^	0.628^∗∗^	0.540^∗∗^	0.344^∗∗^	0.590^∗∗^	0.904^∗∗^	1		
3‐2 Dedication	14.47 (3.27)	0.566^∗∗^	0.481^∗∗^	0.577^∗∗^	0.467^∗∗^	0.314^∗∗^	0.650^∗∗^	0.918^∗∗^	0.776^∗∗^	1	
3‐3 Absorption	12.96 (3.86)	0.484^∗∗^	0.412^∗∗^	0.505^∗∗^	0.433^∗∗^	0.221^∗∗^	0.584^∗∗^	0.905^∗∗^	0.698^∗∗^	0.744^∗∗^	1

^∗∗^ *p* < 0.001.

In addition, to further explore the role of psychological capital in the link between occupational identity and work engagement among professional caregivers, we used AMOS 24.0 software to construct a SEM with occupational identity as the independent variable, work engagement as the dependent variable, and psychological capital as the mediating variable. The model fit indicators are as follows: *χ* ^2^ */*df = 2.834, IFI = 0.931, TLI = 0.918, CFI = 0.930, RMSEA = 0.079 (ideal value: *χ* ^2^ */*df < 3.00; IFI, TLI, CFI > 0.90; RMSEA < 0.08). The results of the SEM analysis indicate that all the fit indicators met the recommended thresholds, the model is considered acceptable, and each path coefficient has reached a significant level, indicating a good fit. The final SEM is illustrated in Figure 1.

**FIGURE 1:**
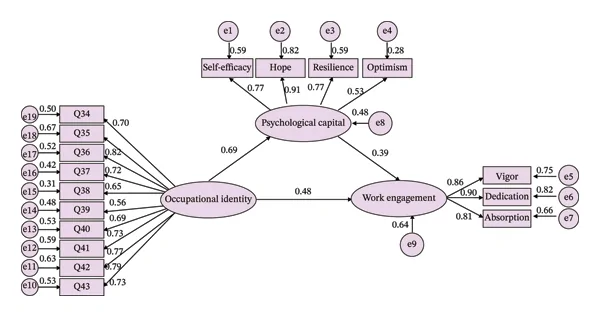
Path diagram for the hypothetical model.

The bootstrap method was used to test the mediating effect by repeating random sampling 5000 times (Table 3). The results indicated that the bootstrap 95% occupational identity intervals of the direct and indirect effects of psychological capital on work engagement did not contain 0. These observations indicated that psychological capital plays a partial mediating role between occupational identity and work engagement, and the mediating effect was 1.147 (95% CI [0.758, 1.682]), accounting for 36.42% of the total effect.

**TABLE 3:** Results for the total, indirect, and direct effects of occupational identity on work engagement, with psychological capital as a mediator (*N* = 296).

Model pathways	Standardized estimate	Unstandardized estimate	*p* value	SE	95% CI		Effect proportion (%)
					Lower	Upper	
Total effect	0.750	3.149	< 0.001	0.310	2.599	3.795	
Indirect effect	0.273	1.147	< 0.001	0.227	0.758	1.682	36.42
Direct effect	0.477	2.002	= 0.001	0.372	1.272	2.731	63.58

Abbreviations: CI, confidence interval; SE, standard error.

To examine whether the inclusion of control variables (age, gender, education, income, tenure, ward, position, and professional training) influenced the hypothesized relationships, we compared two models: one without control variables and one with control variables. The results showed that the model fit indices were comparable between the two models (Table 4). More importantly, the core path coefficients remained stable after controlling for demographic variables (Table 5). The path coefficients of the control variables on work engagement were mostly nonsignificant, indicating that these demographic factors did not substantially confound the relationships among the key variables.

**TABLE 4:** Comparison of model fit indices between Model 1 (without control variables) and Model 2 (with control variables).

Model	*χ* ^2^ */*df	IFI	TLI	CFI	RMSEA
Model 1 (without control variables)	2.834	0.931	0.918	0.93	0.079
Model 2 (with control variables)	2.036	0.929	0.904	0.927	0.059

**TABLE 5:** Comparison of standardized path coefficients between Model 1 (without control variables) and Model 2 (with control variables).

	Model 1		Model 2	
	Standardized estimate	*p*	Standardized estimate	*p*
Psychological capital <‐‐‐Occupational identity	0.693	^∗∗∗^	0.698	^∗∗∗^
Work engagement <‐‐‐Psychological capital	0.395	^∗∗∗^	0.406	^∗∗∗^
Work engagement <‐‐‐Occupational identity	0.477	^∗∗∗^	0.485	^∗∗∗^

^∗∗∗^ *p* < 0.001.

Given the cross‐sectional nature of the data, we also tested a reverse model (Figure 2) to assess alternative explanations. The results show that the fitting index of the reverse model did not meet the acceptable standard (*χ* ^2^ */*df = 4.381, IFI = 0.871, TLI = 0.850, CFI = 0.871, and RMSEA = 0.107), and the key path coefficients were estimated abnormally (standardized coefficients > 1 or negative values), indicating that the data does not support the reverse causal hypothesis. In contrast, the original theoretical model fitted well, and the paths were significant, further verifying the rationality of the hypotheses of this study.

**FIGURE 2:**
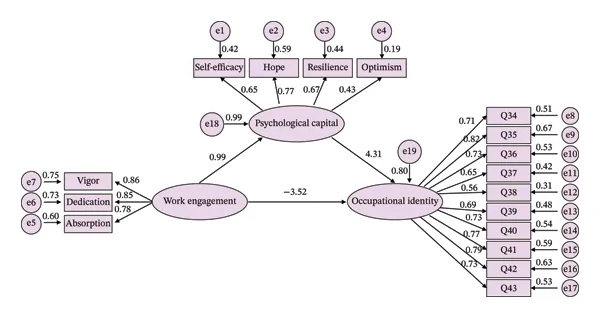
Path diagram for the reverse mediation model.

## 4. Discussion

The professional caregivers were mainly female (80.4%), 89.6% were aged 40–59 years, 87.8% were married, and 79.7% were not well educated (junior high school and below). According to published studies in Chinese journals, most paid caregivers are middle‐aged women with lower educational attainment who were previously farmers from rural areas or laid‐off workers from cities. Our findings are consistent with the previous studies [3, 29, 30]. Perhaps because professional caregiving are frequently characterized as labor‐intensive and emotionally demanding, with core competencies such as turning, feeding, cleaning, and companionship observation often acquired through experiential learning and embodied practice rather than formal academic credentials. Such occupational characteristics have been observed to correspond with workforce profiles that prioritize practical experience over educational qualifications. Concurrently, women in the 45–55 age group may encounter constrained re‐employment opportunities within China’s current labor market. Within this context, traditional Confucian norms that frame caregiving as an extension of women’s domestic roles continue, reinforcing occupational gender segregation where middle‐aged women are stereotypically deemed “naturally suited” for intimate care work.

In our research, tertiary hospitals were selected as the investigation sites because most tertiary referral hospitals provide high‐quality and professional healthcare services to multiple areas located in the city, and these areas exhibit the greatest demand for professional caregivers. Moreover, 28.7% worked in medical wards, and 34.5% worked in surgical wards. In contrast, chronically ill patients and those with disabilities, as well as those after surgery and for patients with critical illness, appear to have a greater demand for professional caregivers, which aligns with the findings of Hui et al. [30]. This observation suggests that the complexity of long‐term care for chronic diseases and disabilities, as well as the high‐risk dependence on postoperative and critical conditions, may be closely associated with the critical role of professional care.

Correlation analysis revealed that the occupational identity of professional caregivers is positively correlated with work engagement and that the dimensions of work engagement are significantly positively correlated with occupational identity.

These results are consistent with the findings of previous research [7, 8, 14]. According to motivation theory, wages, bonuses, benefits, and working conditions are the external motivating factors of one’s work motivation, whereas belonging, respect, achievement, and challenge are the internal motivators of one’s work motivation [31].

Within this theoretical framework, professional identity functions as an internal incentive that may intrinsically unify one’s personal value and meaning with the value and meaning of one’s occupation. As a result, work motivation associated with occupational identity tends to be more conscious and active. Compared with other external stimuli and incentives, occupational identity appears to have a more lasting and stable relationship with work motivation. According to the JD‐R model, occupational identity can be viewed as a personal resource that may help counteract work demands and support work engagement. A stronger professional identity may be associated with a strengthened sense of purpose and motivation, potentially corresponding to greater energy and dedication in caregiving tasks. Therefore, higher levels of professional identity are associated with enthusiasm among professional caregivers, suggesting that those with stronger professional identity may be more actively committed to nursing care work and provide better services for patients. Accordingly, positive feedback from patients may be linked to an enhanced sense of responsibility and accomplishment, potentially fostering a virtuous circle of occupational identity. These findings suggest that strategies aimed at enhancing occupational identity could be valuable for fostering work engagement in this population. Practical steps warranting further investigation may include providing systematic training, clarifying professional value, and establishing fair compensation systems to reinforce professional caregivers’ identification with their role.

There is a significant positive correlation between the occupational identity of professional caregivers and the various dimensions of psychological capital. This observation is similar to the findings of a previous study [19, 32]. Specifically, Qiu et al. [19] reported that individuals with a positive occupational identity tend to invest more time and energy in developing their potential and cultivating a positive self‐perception while actively integrating creativity and motivation into their work. Meanwhile, they also demonstrate remarkable willpower and resilience when confronting difficulties. One possible explanation is that individuals with a high degree of occupational identity may possess a greater understanding of and familiarity with their careers, along with positive expectations regarding possible difficulties encountered in their occupation. Such positive expectations may be associated with more effective adaptation. From the perspective of subdimensions, the understanding and recognition of professional caregivers’ work may be associated with their exploration of work, as well as their professional ability, self‐efficacy, and hope level. External support and positive feedback are also linked to positive psychological states among professional caregivers. For example, the understanding, support, and encouragement of patients may motivate professional caregivers to constantly surpass previous limitations and improve themselves in their work, which correlates with enhanced self‐efficacy and a sense of hope. Similarly, peer guidance, knowledge sharing, and the establishment of excellent role models are associated with professional caregivers’ self‐efficacy and hope levels. Furthermore, family support, colleague support, and a company’s professional and complete resources appear to be related to the resilience of professional caregivers.

There is a significant positive correlation between professional caregiver psychological capital and work engagement. This relationship may be understood through COR theory, which suggests that psychological capital serves as a key personal resource that could be associated with deeper work involvement [33]. The JD‐R model further suggests that psychological capital may play a role in mitigating the resource depletion caused by high job demands, thereby supporting engagement [10]. These findings were consistent with those of previous studies [34, 35]. Higher psychological capital scores were associated with greater degrees of vigor, dedication, and absorption. Individuals with high levels of psychological capital tend to believe that they are competent in their work, hold hope for the future, explore multiple paths to achieve their goals, and demonstrate the ability to recover when encountering difficulties and challenges [34]; consequently, such individuals also tend to exhibit more vigor, dedication, and focus on their work. With respect to the subdimensions of psychological capital, professional caregivers encounter different patients and their families at work and may be exposed to negative emotions. Professional caregivers with a high level of resilience may be better able to disengage from negative emotions and subsequently devote themselves to work with positive emotions. Professional caregivers with a high level of self‐efficacy may have a clearer understanding of their own advantages at work, find suitable and efficient working methods and skills, demonstrate greater flexibility, and experience a higher sense of accomplishment, which is associated with higher work engagement. Optimism may be linked to the recovery of individual physiological function, thus relating to the vitality of professional caregivers’ work. Individuals who are full of hope for the future tend to plan their own future, make unremitting efforts toward this goal, and appear more willing to devote their energy to their work.

The study revealed that the psychological capital of professional caregivers plays a partial mediating role in the relationship between occupational identity and work engagement. That is, occupational identity may be indirectly associated with work engagement through individual psychological capital. First, according to the JD‐R model, the high emotional demands of professional caregiving (such as the empathic stress of the patient’s painful experience) may accelerate resource depletion, whereas professional identity, as an individual resource, could potentially buffer this depletion by being associated with higher levels of psychological capital (such as resilience and hope), thereby sustaining work engagement [10]. Second, COR theory further points out that the stability of occupational identity helps individuals resist the risk of resource loss (such as occupational stigma and psychological threat caused by low social status), whereas the plasticity of psychological capital may form positive feedback through continuous investment through the cycle of resource gain (such as optimism, which is linked to the improvement of problem‐solving ability). Occupational identity reflects an individual’s perception of his own occupation and external recognition, which is closely related to the individual’s emotions and states and consequently the individual’s behavior [8]. A stronger sense of occupational identity is associated with individuals feeling full of optimism and hope for the future, which may relate to a greater tendency to plan their career development path and devote time and energy to their own goals. The recognition of patients and their families is associated with professional caregivers’ self‐efficacy, enabling professional caregivers to recover more quickly and strive for success when facing adversity, setbacks, and difficulties; this is linked to reinvesting in work with vigor and taking the initiative to study work to better complete work.

In practice, strategies to potentially foster the work engagement of professional caregivers may focus on the following aspects. First, regarding the occupational identity of professional caregivers, managers are suggested to provide necessary support, aiming to help employees alleviate conflicts and solve problems collaboratively when they encounter problems or conflicts [36]. Additionally, meeting their reasonable requirements and regularly conducting relevant professional training could be beneficial. Managers should attach importance to the role of professional caregivers in patient care and listen to and affirm appropriate opinions put forward by them. Second, concerning the psychological capital of professional caregivers, managers might provide effective interventions by organizing mindful activities that stimulate positive emotions [37] and delivering psychological counseling lectures. These measures aim to improve their PsyCap level, foster employees’ psychological capital internally, and improve their individual status. Third, in terms of policies, it is recommended that the state enforce the state‐unified vocational qualification certification and industry standards and further improve professional caregiver labor rights, health insurance, occupational injury compensation, and other systems. Furthermore, exploring the path of “professional escort” professionalism could contribute to improving the social recognition of professional caregivers.

The present study was conducted among professional caregivers. However, the identified pattern of associations is not exclusive to the caregiving context. In this pattern, occupational identity was positively related to work engagement through psychological capital. According to the JD‐R model and COR theory, personal resources such as occupational identity and psychological capital have been conceptualized as motivational factors that may be associated with work engagement across diverse occupational settings, particularly in professions characterized by high emotional labor and interpersonal demands. Consistent with this theoretical reasoning, recent evidence from the educational field lends support to the potential generalizability of this pattern of the occupational identity‐work engagement pathway. For instance, Zhai et al. [38] found that professional identity was positively associated with in‐service teachers’ work engagement and psychological capital. This finding appears broadly consistent with the present results, suggesting that occupational identity may function as a general personal resource across human‐service professions, rather than being confined to nursing or caregiving contexts. Nevertheless, it is important to acknowledge the boundary conditions that may shape the strength and nature of this pattern across different occupational fields. Professional caregivers and teachers, while both engaged in helping professions, face distinct occupational demands. Caregivers face more physical care demands and patient‐safety responsibilities, whereas teachers face pedagogical, emotional, and policy‐related demands. These differing demand profiles suggest that while the observed identity‐engagement pattern may be broadly applicable, the specific role of psychological capital within this pattern could vary in magnitude or direction depending on the occupational context.

## 5. Conclusion

The study results support the partial mediating role of PsyCap in the relationship between professional identity and work engagement. Therefore, fostering the occupational identity and personal psychological competence of professional caregivers may contribute to higher levels of work engagement. This study also reveals the mediating effect of PsyCap, suggesting that in addition to providing adequate and professional resources and guarantees for professional caregivers, managers should consider implementing interventions aimed at enhancing the PsyCap of professional caregivers.

### 5.1. Limitations

Some limitations of this study should be acknowledged. First, due to the cross‐sectional design, causal inferences among the variables cannot be drawn. Although the reverse‐model analysis supports the hypothesized relationships, future research should employ longitudinal or intervention designs to track the dynamic changes and causal directions among these variables over time. Second, participants were third‐party contracted professional caregivers recruited via convenience sampling from tertiary hospitals in Sichuan Province. Consequently, the findings are limited to this specific employment and institutional context and should not be generalized to professional caregivers in primary or secondary care facilities, nor to individually practicing caregivers lacking formal institutional affiliation. Thus, the sample may not represent the broader population of Chinese professional caregivers. Third, data were collected from multiple medical institutions, which may introduce clustering effects. The current analysis did not fully account for hospital‐level variations, such as differences in organizational culture and management style. Future research should employ multilevel modeling (e.g., HLM) to examine cross‐level moderating effects of organizational factors on the relationship between individual psychological capital and work engagement. Fourthly, the cross‐sectional, self‐report design renders the findings susceptible to CMB. To address this more rigorously, future studies should go beyond procedural and statistical remedies by employing multisource designs (e.g., obtaining supervisor ratings of employee engagement), temporally separating of measurements, or incorporating objective behavioral indicators to further validate the present findings.

## Funding

The authors have nothing to report.

## Conflicts of Interest

The authors declare no conflicts of interest.

## Supporting Information

Additional supporting information can be found online in the Supporting Information section.

## Supporting information


**Supporting Information** STROBE checklist.

## Data Availability

The processed data can be obtained from the corresponding author upon reasonable request.
